# Choosing what is left: the spatial structure of a wild herbivore population within a livestock-dominated landscape

**DOI:** 10.7717/peerj.8945

**Published:** 2020-04-08

**Authors:** Milagros Antún, Ricardo Baldi

**Affiliations:** 1 Grupo de Estudio de Mamíferos Terrestres, Instituto Patagónico para el Estudio de los Ecosistemas Continentales (IPEEC) - CONICET, Puerto Madryn, Chubut, Argentina; 2 Facultad de Ciencias Naturales y Ciencias de la Salud, Departamento de Biología y Ambiente, Universidad Nacional de la Patagonia San Juan Bosco, Puerto Madryn, Chubut, Argentina; 3 Programa de Maestría en Conservación de la Biodiversidad, Departamento de Ecología, Genética y Evolución, Facultad de Ciencias Exactas y Naturales, Universidad de Buenos Aires, Buenos Aires, Argentina

**Keywords:** Distribution and abundance, Lama guanicoe, Habitat selection, Spatial models, Sheep ranching, Anthropic factors, Wild and domestic ungulates, Península Valdes, Patagonia

## Abstract

Shrublands and grasslands comprise over 30% of the land surface and are among the most exploited ecosystems for livestock production. Across natural landscapes, the distribution and abundance of wild herbivores are affected by interspecific competition for foraging resources, hunting and the development of infrastructure among other factors. In Argentine Patagonia, the abundance of domestic sheep grazing on native vegetation outnumbers the widely distributed guanaco (*Lama guanicoe*) and sheep ranching monopolizes the most productive lands. In this work, we aimed to assess the spatial variation in the abundance of guanacos in Península Valdés, a representative landscape of Patagonia, investigating the incidence of natural and human-related factors. We conducted ground surveys during the austral autumn in 2017 totaling 383.4 km along areas with and without sheep ranching. We built density surface models to account for the variation in guanaco abundance and obtained a map of guanaco density at a resolution of 4 km^2^. We estimated an overall density of 11.71 guanacos.km^−2^ for a prediction area of 3,196 km^2^, although the density of guanacos tripled in areas where sheep ranching was terminated (in around 20% of the surface of Península Valdés) compared to areas with sheep. Guanacos were more abundant at lower values of primary productivity and sheep stocking rates and further from inhabited ranch buildings, suggesting competition with sheep and conflict with humans. Although guanacos selected open, grass-dominated habitats across sheep-free sites, fences dividing properties and paddocks played a significant role in the spatial structure of their population in Península Valdés affecting negatively the abundance of guanacos. Our results indicate that actions to improve habitat connectivity for guanacos, favor the coexistence among guanacos and sheep ranching, and promote responsible human activities and attitudes towards wildlife are needed.

## Introduction

The study of wild species distribution and abundance allows exploring the mechanisms and processes that operate in the selection of habitats and the factors related to the persistence of populations. While variation in population density has been described in terms of combinations of physical and biotic conditions across a species’ distributional range (Brown, 1984), within-population changes in local abundance are the result of habitat selection by individuals (Rosenzweig, 1981; Morris, 2003; Bjørneraas et al., 2012). The spatial structure of wild populations depends largely on biological requirements, species life history (Henle et al., 2004; Ewers & Didham, 2006; Prugh et al., 2008), capacity of dispersal, intra and interspecific interactions and evolutionary processes (Peres-Neto & Legendre, 2010). However, it is known that human activities affected the distribution and abundance of many wild species over time. Large, wild herbivores were important sources of food and clothing for humans thousands of years ago (Anderson, 1985; Roth & Merz, 1997). The subsequent expansion and demographic growth of human populations imposes new uses of wild species that included domestication, for example, of the sheep (*Ovis orientalis aries*) from the mouflon (*Ovis orientalis orientalis*) among others (Olsen, 1990).

Production systems based on the introduction of domestic livestock—mainly sheep, cattle and goats (FAO, 2017)—in natural landscapes resulted in changes in the perception of wild species and their value as a resource for humans. Moreover, millions of domestic herbivores grazing on the native vegetation affected the structure and functioning of a wide range of ecosystems. Arid shrublands and grasslands are among the most extensively exploited ecosystems in the world for livestock production, comprising over 30% of the land surface (Adeel et al., 2005; Millenium Ecosystem Assessment, 2005) and supporting a wide range of ecosystem services (Mortimore, 2009). Across the rangelands, or natural areas used by people to graze their livestock, wild herbivores have been largely affected by habitat degradation due to overgrazing, competition for foraging resources, hunting and the development of infrastructure, among other threats (Primack, 1998; Montgomery, 2007; Ellis & Ramankutty, 2008). Wild ungulates and introduced domestic species of similar body size and digestive system (e.g., similar sized ruminants) are likely to have similar nutritional requirements (Jarman, 1974; Illius & Gordon, 1992) hence increasing the potential for interspecific competition (Schoener, 1974; Belovsky, 1986). Frequently, spatial segregation is the result of wild ungulates moving across modified landscapes towards areas that usually do not represent their habitat preferences (Prins, 2000) but restrictions imposed by interspecific competition with livestock and conflict with human activities.

In arid Patagonia, extensive sheep ranching started late in the 19th century when European colonists settled in the region, reaching 22 million heads within 40 years (Soriano & Movia, 1986). Sheep grazing affected vegetation structure and primary production by decreasing the proportion of plant species of a higher foraging value (Adler et al., 2005; Chartier & Rostagno, 2006; Bisigato, Laphitz & Carrera, 2008; Cesa & Paruelo, 2011), while accelerating desertification processes (del Valle et al., 1998). The massive introduction of the domestic sheep affected the numbers and distribution of guanacos, the only large, wild ungulate extensively distributed across the arid lands of Patagonia. Over hunting, range degradation and interspecific competition with sheep have contributed to the guanaco’s demise (Raedeke, 1979; Franklin, 1982; Cunazza, Puig & Villalba, 1995; Baldi, Albon & Elston, 2001; Baldi et al., 2004). Although still abundant and classified as of “Least Concern” by the IUCN, the guanaco occupies around 30% of its original range and 80% of its global population remains in Argentina, particularly throughout Patagonia (Baldi et al., 2016).

Guanacos are generalist herbivores including grasses and shrubs in their diet (Pelliza-Sbriller et al., 1997; Puig, Videla & Cona, 1997). Sheep diet composition is highly similar to that of guanacos and it was estimated that only 17 plant species made up around 80% of the diet of both herbivores, resulting in a high potential for interspecific competition (Baldi et al., 2004). Across the region, guanaco abundance has been found to be negatively related to sheep numbers. Evidence of spatial and temporal segregation due to interspecific competition has shown that guanacos occupied marginal habitats after the most productive sites were monopolized for sheep ranching (Baldi, Albon & Elston, 2001; Pedrana et al., 2010). However, socio-ecological systems are complex and tend to be dynamic over time and space (Reynolds et al., 2007). Sheep numbers declined in Patagonia during the last 30 years due to a combination of economic and ecological factors (Aagesen, 2000; Coronato et al., 2016), which resulted either in ranches with low sheep stocking rates or properties where the activity was terminated but infrastructure—such as ranch buildings and outstation, permanent water sources and fences—remain.

The decrease of both sheep stocking rates and spatial extent of ranching activities has been associated to an increase in the numbers of some populations of guanacos in Patagonia (Novaro, Baldi & Antún, 2019). This brings the opportunity to explore to what extent the processes and factors related to guanaco population demise are operating today and how are guanaco populations responding to recent changes across modified landscapes. We investigated the incidence of natural and human-related factors on the spatial variation in the abundance of guanacos in Península Valdés, a representative landscape of the arid Patagonia where sheep were introduced in the 1880s, but removed from nearly 20% of the area during the last 20 years (Baldi et al., 2017). We hypothesized that (1) interspecific competition with sheep still plays a key role in shaping the spatial structure of the guanaco population in PV; (2) the conflict with humans influences the variation in the abundance of guanacos in areas where sheep ranching takes place; and (3) the importance of natural factors will become evident at sites without sheep and threats imposed by human presence, reflecting what is left available to guanacos in terms of habitat to select. According to our hypotheses, we predict that (1.1) the abundance of guanacos will decrease while sheep stocking rate and (1.2) primary productivity increase; (2.1) guanacos will be less abundant in the proximity of infrastructure elements such as inhabited ranch buildings and fences, but (2.2) will be favored by the proximity to permanent water sources for sheep. In areas where the activity has ceased, we predict that (3.1) plant productivity and (3.2) open, grass-dominated sites in flat areas will be associated with increased guanaco numbers.

## Materials and Methods

The present work is a non-invasive study, conducted through the observation of animals by means of binoculars. Permission for the research was given by the Direction of Conservation and Protected Areas, and the Direction of Wildlife of the Chubut Province (DF & FS-SSG, Permit 69/2016).

### Study area

**Figure 1:**
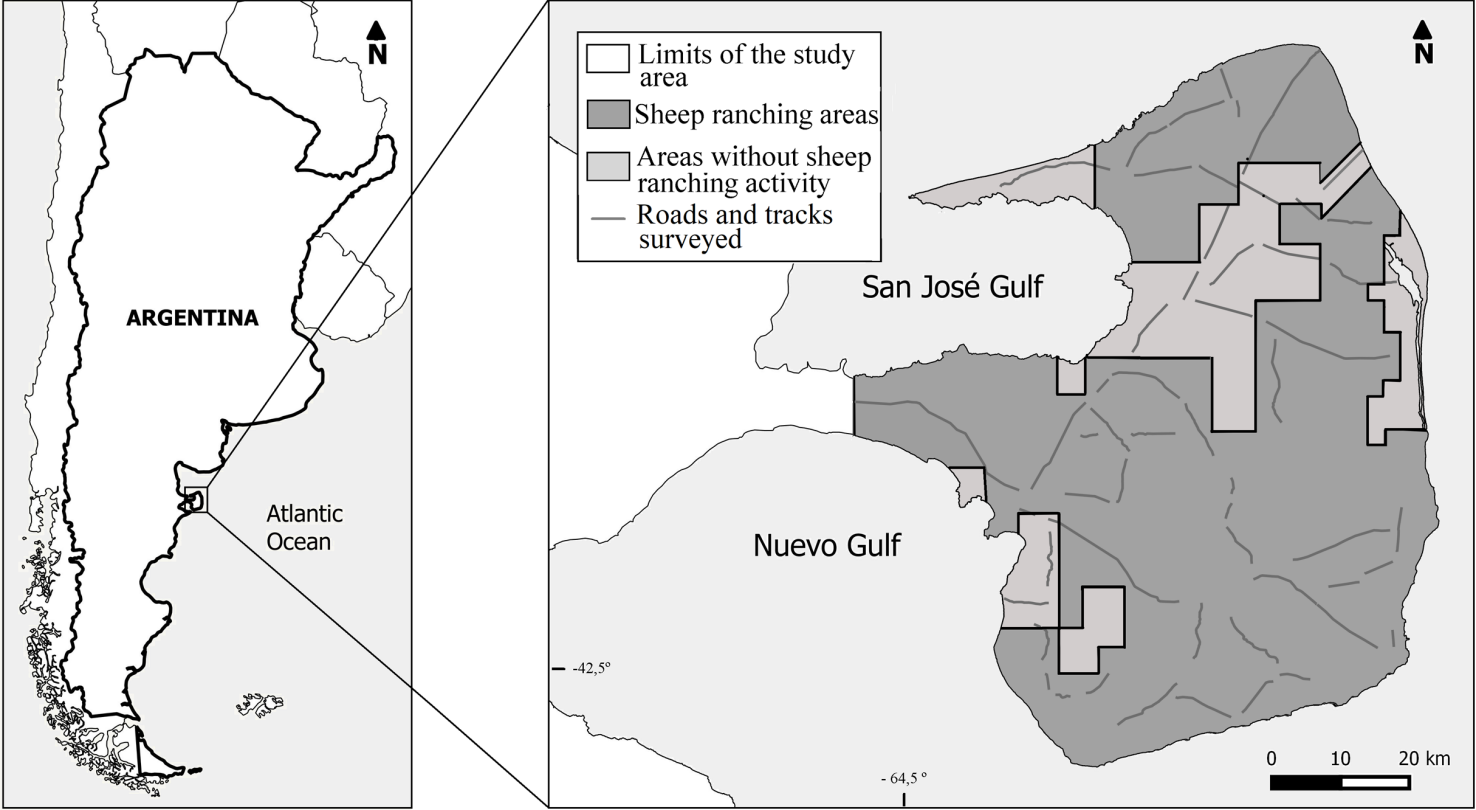
Location of Península Valdés and distribution of the survey transects inside the study area. Sheep ranching areas (SHEEP) are represented in dark grey while the areas where the activity has ceased (NOSHEEP) are in light grey.

The study was carried out in Península Valdés (PV, Fig. 1), a provincial protected area and also a UNESCO World Heritage Site located in the Argentine Patagonia. The area presents a temperate semi-arid climate with a mean annual temperature of 13.6 °C (Barros, 1983), and an average annual rainfall of about 230 mm with a high interannual variation (100–300 mm; Coronato, Pessacg & Alvarez, 2017). The vegetation is represented by the southern Monte and the northern Patagonian Phytogeographic Provinces (León et al., 1998). Shrubs and grass-shrubs steppes dominate northern and central PV with a vegetation cover that varies between 40% and 60%, while grass steppes predominate in the southern part of the area with an average cover of 70% (Fig. 1; Bertiller et al., 2017). The most abundant shrub species are *Chuquiraga avellanedae* and *Chuquiraga erinacea*, while the predominant perennial grasses are species with high forage value as *Nassella tenuis, Piptochaetium napostaence, Poa ligularis* and *Poa Lanuginosa* (Bertiller et al., 2017).

Extensive sheep ranching for wool production predominates the surface of PV (SHEEP areas; Fig. 1), which is divided by fences into more than 60 properties. Each ranch is subsequently fenced into a series of irregular paddocks of variable size (100–2,500 ha) where the sheep graze on the native vegetation. There is usually one building per ranch permanently occupied by a rural worker. Although there are no permanent residents in areas where sheep ranching has ceased (NOSHEEP areas; Fig. 1), fences and other infrastructure such as water tanks and windmills remain in place.

### Field surveys

We conducted ground, line transect surveys (Buckland et al., 1993; Laake et al., 1993) of guanacos at the beginning of the autumn in 2017, totaling 383.4 km surveyed along secondary dirt-roads and tracks (average transect length: 7 km). Surveys were conducted as previously described in Antún et al. (2018).

### Selection of predictor variables

To test our hypotheses, we identified natural and anthropic variables as potential predictors of *L. guanicoe* abundance (Table 1). As a correlate of primary productivity, we calculated the mean Normalized Difference Vegetation Index (NDVI; Travaini et al., 2007) for the spring-summer season of 2016–2017 (from September 21st to March 21st). As described in Antún & Baldi (2019) we calculated the CV of the NDVI to account for variation in vegetation physiognomy, and found that it was larger across shrub steppes than in mixed and grass steppes where grasses with high forage value predominate (see Supplemental Information 1). The variables derived from the NDVI were based on MODIS MOD13Q1 satellite images of 250 m spatial resolution available at https://lpdaac.usgs.gov. Altitude values were obtained from the Digital Elevation Model for South America (resolution of about 220 m) at https://lta.cr.usgs.gov/SRTM1Arc. Subsequently, we calculated the CV of the altitude as an estimate of terrain irregularity, relevant in terms of early detection of predators by guanacos (Bank et al., 2002; Pedrana et al., 2010). The current sheep stocking per paddock was obtained by consulting landowners and rural workers between 2015 and 2017. Data on the location of ranches, permanent water sources, and wire fences delimiting the paddocks were available from the database at our institute but checked and updated in the field between 2013 and 2017 (Antún, 2018). Additionally, we included latitude and longitude as geographic variables. Although the geographical predictors do not have ecological significance, they could account for the effect of other variables not available to be included (Travaini et al., 2007) such as the spatial variation in precipitation (Coronato, Pessacg & Alvarez, 2017; Antún et al., 2018; Table 1). We obtained the values for each variable using the QGIS Open Source Geographic Information System (QGIS Development Team, 2016) and packages reshape2 version 1.4.2 (Wickham, 2007), raster version 2.5.8 (Hijmans et al., 2016) and ggplot2 version 2.2.1 (Wickham & Chang, 2016; R software, version 3.2.1, R Development Core Team, 2015). The range of values of each variable across the study area was included as far as possible in the surveyed tracks. Multicollinearity in predictor variables could make it difficult to separate the effects on the response variable and to compare alternative models (Lennon, 1999), thus we evaluated the collinearity between pairs of covariates taking the values measured at each segment (see below). We considered two predictors not to be collinear when Pearson’s correlation coefficients were <0.7. The variables CV of NDVI and geographic latitude showed collinearity (|*r*| > 0.7). Thus, we kept the former as we considered its ecological significance and possible effect on the spatial structure of the guanaco.

**Table 1:** List and description of all the variables used.

Variable type	Name of the variable	Description
Natural	Mean NDVI	Mean Normalized Difference Vegetation Index for the spring-summer season of 2016–2017. Used as a correlate of plant productivity
	CV NDVI	Coefficient of variation of the Normalized Difference Vegetation Index from 2010 to 2014. Used as a correlate of vegetation physiognomy
	CV altitude	Coefficient of variation of the mean altitude. Used to describe the topography of the terrain
Anthropic	Ranch distance	Distance to the nearest ranch building in meters
	Sheep stocking	Sheep stocking rate (sheep.km^−2^) per paddock
	Water distance	Distance to the nearest, permanent water source in meters. Troughs for the sheep are either associated to windmills or tanks
	Fence distance	Distance to the nearest fence in meters
Geographical	Longitude	Longitude projected into meters using Universal Transverse Mercator zone 20. Used as a correlate of the precipitation regime
	Latitude	Latitude projected into meters using Universal Transverse Mercator zone 20. Used as a correlate of the precipitation regime

### Estimating the detection function

Using standard distance sampling methodology (Buckland et al., 1993), we fitted detection functions to account for the probability of detecting guanacos for the whole of PV, and for SHEEP and NOSHEEP areas. We evaluated the half-normal, hazard-rate and uniform functions as candidate detection functions (Thomas et al., 2010). The final functions were selected following the procedure detailed in Antún et al. (2018). All analyses were performed using the “Distance” package version 0.9.6 (Miller, 2017) for R.

### Density surface model (DSM)

Each transect line was divided into smaller segments of 1.8 km in length and 2 km wide (Miller et al., 2013; Antún & Baldi, 2019), totaling 213 segments in all the study area of PV, 164 located in SHEEP and 49 in NOSHEEP. Subsequently, each observation was assigned to its corresponding segment according to its location. The size of the segment was defined according to the information available for the population of guanacos of PV (a sedentary population with an average home range of 5.5 km^2^; Burgi, 2005), the detection functions and the length of the transects (Schroeder et al., 2014). When the detection function did not include other covariates than distance, the probability of detection was constant for all segments, and therefore we modeled the species abundance per segment using generalized additive models (GAMs; Wood, 2006) with the “count method” (Hedley & Buckland, 2004). Contrary, when the detection function included the group size as a covariate, the abundance was estimated by GAMs but with the Horvitz–Thompson-like estimator (Hedley & Buckland, 2004). The abundance in each segment was estimated including the probability of detection and described as the sum of smooth functions of uncorrelated predictor variables measured at the segment. Restricted Maximum Likelihood (REML) was used for smoothness selection of all the GAMs performed (Reiss & Ogden, 2009; Wood, 2011). The concurvity (the non-linear multicollinearity) could lead GAMs to produce unstable or even wrong estimates of the covariates’ functional effects (Peng, Dominici & Louis, 2006; Gu, Kenney & Zhu, 2010). Consequently the concurvity degree of the smoothing terms (Wood, 2006) was evaluated after fitting the models (Miller et al., 2018) and so we guarantee that any smoothing term was approximated by one or more of the other smoothing terms in the model. Following Miller et al. (2013) for each GAM we explored three response distributions including: Tweedie, negative binomial and quasi-Poisson. For each distribution we built a “base model” and considered all the covariates as univariate smooths. We performed the covariate selection in each base model by removing the non-significant covariates (with approximate *P*-values > 0.001; Marra & Wood, 2011). We obtained three models as final candidates for each area. Finally we selected the best fitting DSM for the whole PV, SHEEP and NOSHEEP areas based on the inspection of residual plots (Miller et al., 2013). The concurvity measures were very small in all the models evaluated, suggesting negligible concurvity (Wood, 2006). Residual autocorrelation was checked as previously described in Antún & Baldi (2019). Models were fitted using the ‘dsm’ package version 2.2.16 for R (Miller et al., 2018; modeling procedure can be checked at http://github.com/DistanceDevelopment/dsm).

### Abundance estimation

Our study area was split into 4 km^2^ cells. Subsequently, we excluded zones with values beyond the surveyed range of the significant covariates and areas adjacent to the coastal limits of the study site or inside the salt pans as they represent marginal habitat of the study area that have not been surveyed. Then, we obtained a prediction surface of 3,196 km^2^ for all the PV area, 2,616 km^2^ for SHEEP and 580 km^2^ for NOSHEEP areas. Finally, we predicted the number of animals for each cell according to the selected DSMs and subsequently obtained an overall estimate of abundance in each area.

## Results

**Figure 2:**
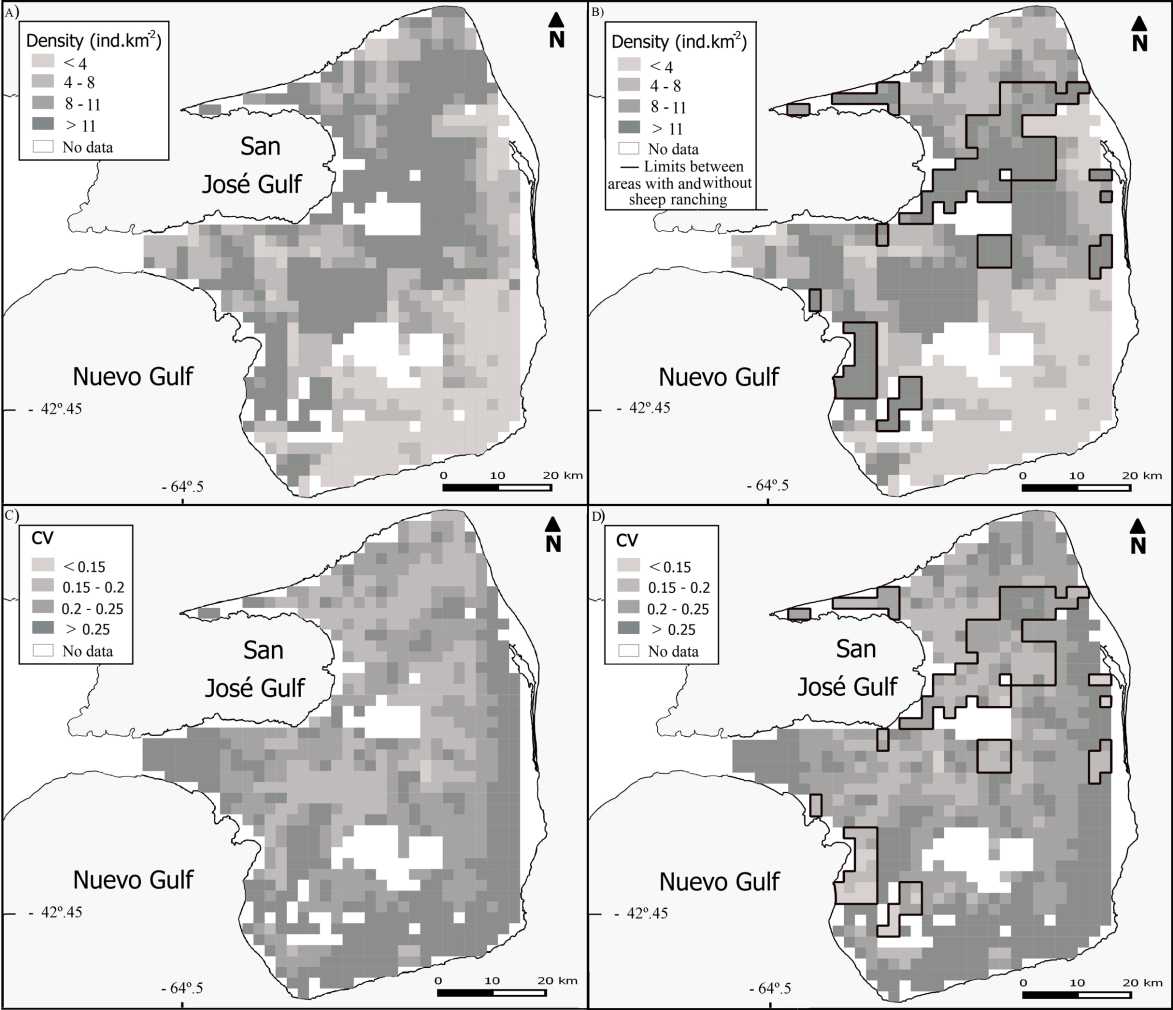
Spatial variation in the abundance of *Lama guanicoe*. Maps of population densities according to (A) the whole PV model and (B) independent models for areas with and without sheep ranching activity (SHEEP and NOSHEEP respectively). Distribution of the coefficient of variation (CV) according to (C) whole PV model and (D) models applied to SHEEP and NOSHEEP areas. Black lines show the limits of NOSHEEP areas.

We recorded 2.09 observations of guanacos per km surveyed across PV, although the average 1.47 observations.km^−1^ for SHEEP areas was markedly lower than 4.43 observations of guanacos per km surveyed in NOSHEEP areas. The best fitting selected models included the tweedie distribution with a logarithmic link function. The average density estimated for the whole PV was 11.71 guanacos.km^−2^ (CV = 8%), although in SHEEP areas the density of guanacos was almost three times lower than in NOSHEEP areas (Table 2; Table S1). Regarding the spatial variation, the highest guanaco densities tended to occur in central and northeastern PV (Fig. 2A). However, densities higher than average concentrated in NOSHEEP areas while the lowest densities of guanacos were estimated and where sheep ranching takes place (Fig. 2B). Uncertainty associated with the density estimates was moderate (Figs. 2C and 2D). The detection functions selected were the half-normal with the truncated data for PV, the hazard-rate using group size as a covariate for SHEEP areas, while for NOSHEEP areas the uniform function with the truncated data (Fig. S1).

**Table 2:** Density of guanacos and significant variables explaining its spatial variation for the whole study area (PV model), and for the areas with and without sheep ranching (SHEEP model and NOSHEEP model respectively).

	PV model	SHEEP model	NOSHEEP model
Average density (guanacos.km^−2^)	11.71	8.02	22.76
Coefficient of variation (%)	8	11	18
Significant variables (*P* < 0.001)	NDVI Mean*	NDVI Mean*	NDVI CV**
	Ranch distance**	Ranch distance**	Fence distance**
	Sheep stocking rate**	Sheep stocking rate**	
	Longitude*	Longitude*	

**Notes:**

* *P* < 0.001.

** *P* < 0.0001.

Statistically significant variables (*P*-values < 0.001) included in the best-fit model for PV and SHEEP areas were the same: NDVI Mean, ranch distance, sheep stocking rate and longitude (Table 2). Whereas, the significant variables explaining the variation in guanaco abundance in NOSHEEP areas model were the coefficient of variation of the NDVI and the distance to the nearest fence (Table 2).

**Figure 3:**
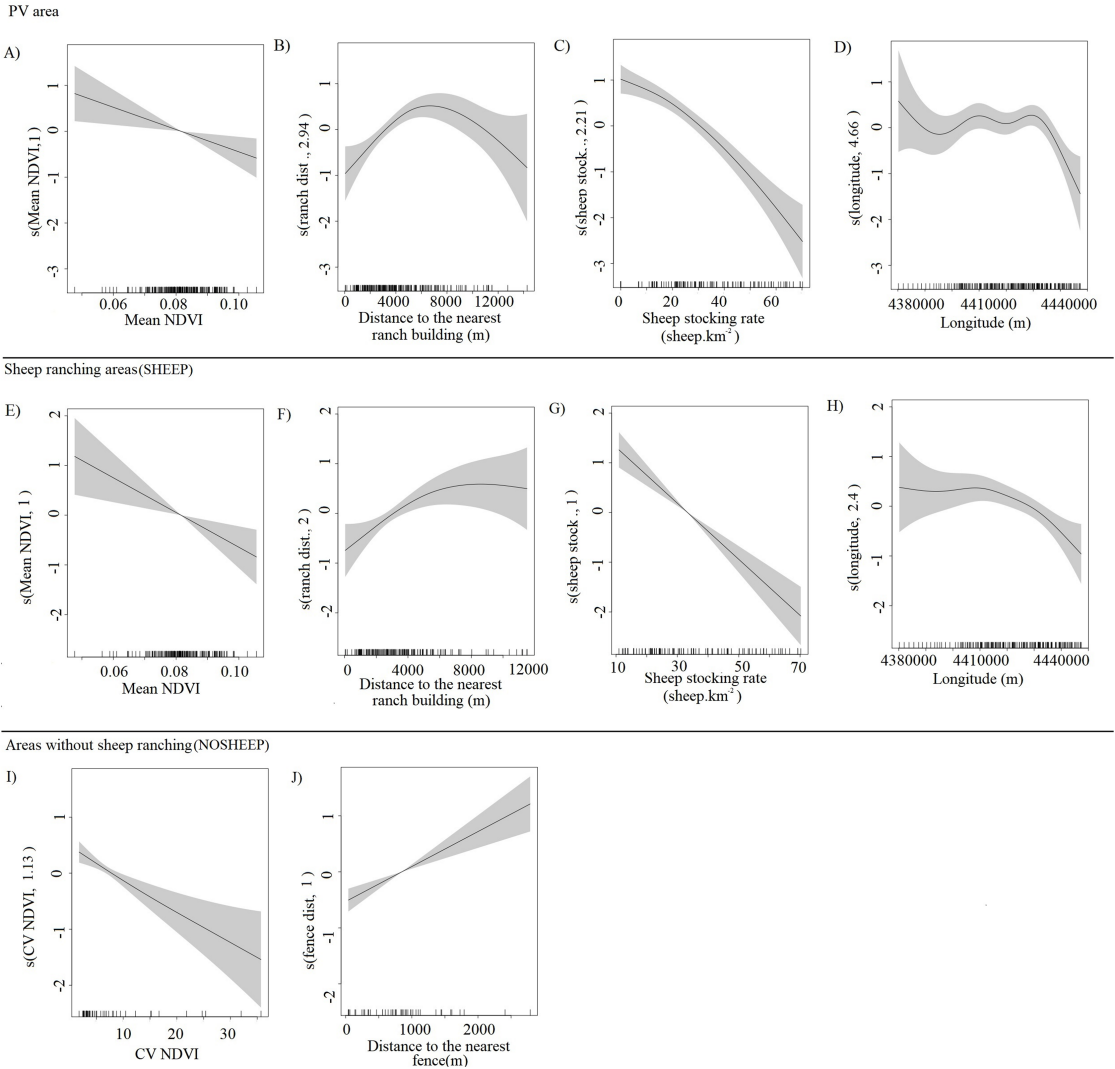
Partial effects of the significant predictors on the abundance of *Lama guanicoe* according to the best-fit model for each area analyzed. Whole Peninsula Valdés area (A–D), sheep ranching areas (SHEEP; E–H) and areas where the activity has been ceased (NOSHEEP; I and J). The solid lines represent the estimated smoothing terms (s) of each predictor and the gray shading represents 95% confidence intervals for the mean effect. The rug ticks at the bottom of the plot indicate the coverage of the range of values of each variable in the survey area. The number in brackets in each “s” gives the effective degrees of freedom (a measure of flexibility) of each term. The *y*-axis is on the scale of the link function.

The abundance of guanacos showed both linear and non-linear relationships with the different predictors (*effective degrees of freedom* = 1 and >1 respectively), and the confidence intervals of the smooth functions tended to be wider where the range of the predictor variables had reduced survey coverage (Fig. 3). Both for the whole PV and for SHEEP areas only, the number of guanacos decreased as the NDVI increased (Figs. 3A and 3E), while guanacos were more abundant as the distance from ranch buildings increased until 6,000 m (Figs. 3B and 3F). Sheep stocking rate had the strongest, negative effect in guanaco abundance as shown by PV and SHEEP models (Figs. 3C and 3G). Geographic longitude showed a weak significant effect on guanaco abundance in both PV and SHEEP areas, restricted to the highest values corresponding to the eastern side of the study area (Figs. 3D and 3H). In NOSHEEP areas, guanacos tended to be more abundant at low values of the CV of the NDVI, but as the predictor value increased its effect became weak (Fig. 3I). Also, in NOSHEEP the location of fences had a strongly significant effect on the abundance of guanacos, which increased in numbers with the distance from the nearest fence (Fig. 3J). A small amount of unmodelled correlation in residuals (<0.2) was observed between adjacent segments in the fitted model of PV and SHEEP areas but we assumed that it did not affect the explanatory capacity of the models (Dellabianca et al., 2016), while in the NOSHEEP model no spatial autocorrelation of residuals was observed. The deviance explained by the selected DSMs was 50.9% in PV, 38% in SHEEP and 47.1% in NOSHEEP areas.

## Discussion

Our results show that the spatial pattern of guanaco abundance is strongly influenced by sheep ranching activity in Península Valdés and guanaco density in areas without sheep almost triples compared to those where both species are present. Although natural factors explaining the spatial variation in the abundance of guanacos emerge in areas without livestock, the influence of the anthropogenic landscape is still significant.

Across the whole Península Valdés, guanacos increased their abundance where sheep densities were lower and decreased at high NDVI values, which is consistent with the hypothesis of interspecific competition with sheep for forage resources (Baldi, Albon & Elston, 2001). Also, guanacos avoided the proximity to inhabited buildings, indicating that the conflict with humans influences their abundance. The same factors were significant to explain the variation in the abundance of guanacos after our model was restricted to SHEEP areas (around 80% of the study area, see Fig. 1), thus influencing the spatial structure in the whole landscape. However, when examining NOSHEEP areas we found that guanacos were more abundant in open, grass dominated steppes and far from fences. Therefore, guanacos select habitats only in areas where sheep ranching ceased, although remaining infrastructure such as fences affected their spatial variation in abundance.

### Competitive interactions and conflict with humans

As intermediate feeders (sensu Jarman, 1974) able to forage on grasses and dicotyledonous plants, guanacos and sheep largely overlap their diets increasing the potential for interspecific competition (Baldi et al., 2004). Spatial and temporal segregation between species have been previously recorded at different sites and guanacos were less abundant where the proportion of preferred plant species was higher, supporting the hypothesis of competition for forage (Baldi, Albon & Elston, 2001; Burgi et al., 2011). Changes in the abundance and displacement of wild ungulates in presence of livestock have been documented across a range of socio ecological systems such as African savannas (Fritz, Garine-Wichatitsky & Letessier, 1996; Hibert et al., 2010), North American grasslands (Loft, Menke & Kie, 1991) and Trans Himalayan rangelands (Mishra et al., 2004) among others.

Although the observed patterns in this study—such as contrasting densities of guanacos in sympatric and allopatric conditions in relation to sheep—are consistent with the hypothesis of interspecific competition, the influence of human presence is significant. Other studies reported that the probability of finding guanacos decreased as human presence increased (Pedrana et al., 2010; Schroeder et al., 2014), reflecting the threats imposed by chasing and hunting and therefore the additional limitations to select habitats. Although Península Valdés is a protected area, guanacos are still harassed by landowners and rural workers due to the conflict with sheep ranching for forage resources and water (Antún, 2018). To what extent is the observed pattern of variation in guanaco abundance the result of competitive interactions with sheep or human harassment? In this study, the predictive variables “sheep stocking rate” and “distance to the nearest ranch building” allowed us to assess their effects independently (see collinearity and concurvity in subsections *Selection of predictor variables* and *Density surface model*). Additionally, our results showed that the effect of sheep stocking rate is stronger than that of the distance to inhabited buildings (see Fig. 3), but we cannot rule out other human-related variables potentially associated with sheep stocking rate (e.g., hunting pressure). However, as some landowners are currently implementing protocols of coexistence among sheep ranching and wildlife populations, there is an opportunity to conduct studies under controlled conditions of no hunting or harassment to understand the mechanisms underlying the competitive interactions between guanacos and sheep.

### What’s left to choose?

Our results suggest that guanacos would still be able to select open habitats—in terms of vegetation physiognomy—within a modified landscape where human activities predominate. Across sheep-free areas where the average density of guanacos almost triples compared to the rest of PV, we found that they were more abundant at grassland and mixed steppe habitats. Guanacos have been described as “intermediate feeders” with perennial grasses and evergreen shrubs accounting for up to 60% of their diet (Bonino & Pelliza-Sbriller, 1991; Puig, 1995; Puig et al., 2001; Somlo, 1997; Baldi et al., 2004). Also, open habitats favored the formation of larger groups of guanacos compared to tall shrub dominated habitats. Marino & Baldi (2008) found that group size was negatively related to individual time spent vigilant, while individual time invested in foraging increased. Therefore, the availability of mono and dicotyledonous plants and the benefits derived from grouping behavior can be influencing selection of mixed steppe habitats by guanacos across NOSHEEP areas.

The average density of guanacos in areas where sheep were removed was almost three times higher than in areas where the activity continued. Although guanacos selected habitat types as shown above, we found a significant influence of the remaining fences on their spatial variation in abundance across NOSHEEP areas. In Península Valdés, fences dividing properties and paddocks add up 2,135 km forming 284 polygons inside our prediction area of 3,196 km^2^ (Nabte et al., 2013; Antún, 2018). The occurrence of guanaco seasonal migration has been associated to the absence of fences and other anthropogenic barriers in the Payunia Reserve in NW Patagonia (Schroeder et al., 2014). In contrast, movements of radio-collared guanacos were found to be limited by fences, which added up 175 km within a 400 km^2^ area comprised by private properties in NW Patagonia (Rey, Novaro & Guichón, 2012; Carmanchahi et al., 2015). Additionally, entanglement was reported to account for up to 6.7% of total annual mortality estimated for the same population, affecting mainly juveniles (Rey, Novaro & Guichón, 2012). Whether or not fences affect the ability of guanacos to move around NOSHEEP areas in Península Valdés, hence imposing further restrictions to habitat selection, is a matter requiring further investigation. Detailed studies on guanaco individual movement can throw light on the processes and mechanisms underlying habitat selection and its relationship with productivity gradients and barriers imposed by human activities.

### Methodological aspects

The use of density surface models allows to evaluate the spatial variation and estimate the abundance of populations for either a whole area or any sub-region. The combination of distance sampling methods with spatial modeling techniques provide unbiased estimates of abundance independent of the sampling design (Hedley & Buckland, 2004). However, the distribution of the available roads and tracks might limit the range of values of a given variable surveyed and therefore the extent of the prediction area. In our study area the transects surveyed covered most of the range of each significant variable, thus the extent of the prediction is nearly the whole study area (Fig. 2).

### Future prospects

It has been argued that high-density populations of guanacos in Patagonia occur on the few protected areas or on land that is abandoned or where sheep ranching was terminated (Novaro, Funes & Walker, 2000; Baldi et al., 2010). Despite facing threats, the number of guanacos in Península Valdés increased markedly during the last 25 years. The average density reported in this study (11.71 ± 0.99 guanacos.km^−2^) is markedly higher than previous estimates for Península Valdés, that turned out to be as low as 1 guanaco.km^2^ (Baldi, Campagna & Saba, 1997), and similar to the density reported for another large protected area, the Payunia Reserve in NW Patagonia (12.28 ± 3.69 guanacos.km^−2^; Schroeder et al., 2014). Although 80% of the land in Península Valdés is still devoted to sheep ranching, the overall stock is probably the lowest in decades (Evolución Existencia de Ganado Ovino 2005–2014, 2016). Both the consolidation of Península Valdés as a protected area, declared as World Heritage Site in 1999, and the decline of sheep ranching could have contributed to the recovery of the population of guanacos. However human—carnivore conflict persists and pumas (*Puma concolor*) are still chased and killed by rural workers, the occasional presence of the native predator of guanacos has been reported back in PV during the last few years (D’Agostino, 2018). If management actions oriented to the coexistence of wildlife and human activities are implemented, we believe there is an opportunity to restore functional populations of native herbivore and carnivore species in Península Valdés.

## Conclusions

Human activities related to sheep ranching for wool production shape the spatial structure of the guanaco population across the Península Valdés landscape. Habitat selection is highly restricted by sheep numbers and human presence, resulting in the guanacos selecting less productive habitats or sites where sheep ranching was terminated. Across sheep-free areas, guanacos showed a positive association to open, grass dominated communities, but still their spatial variation in abundance was affected by the proximity to wire fences dividing properties and paddocks within ranches. Using a single Density Surface Model we (i) described the spatial variation in the abundance of guanacos at a higher resolution than previous estimates for Península Valdés; (ii) identified the main variables explaining the spatial structure in different contexts; and (iii) demonstrated that human-related effects such as infrastructure are still significant even after sheep ranching ceased. This approach can contribute to assess the population abundance and distribution of guanacos elsewhere across its range, by combining the well-known distance sampling survey method with spatial modeling. The abundance of guanacos reported here is among the highest estimated across their range, probably due to the fact that Península Valdés is a World Heritage Site and also to the decreasing trend in sheep numbers. However, it is necessary the implementation of conservation and management actions to favor coexistence among guanacos and sheep ranching, improve habitat connectivity for guanacos, and promote responsible human activities and attitudes towards wildlife. Future research should focus on the spatial dynamics of guanacos—both individual and groups—to understand the underlying mechanisms operating on the observed patterns.

## Supplemental Information

10.7717/peerj.8945/supp-1 Supplemental Information 1 Analysis of the variation coefficient of the Normalized Vegetation Index (CV NDVI).

10.7717/peerj.8945/supp-2 Supplemental Information 2 Comparison of average densities estimated by each model (PV, SHEEP and NOSHEEP) for the whole PV and in areas with and without sheep ranching.

10.7717/peerj.8945/supp-3 Supplemental Information 3 Detection function modeling.

10.7717/peerj.8945/supp-4 Supplemental Information 4 Effect of significant smooth terms on the response variable (expressed as number of guanacos per segment), according to the selected models for Península Valdés and the areas with and without sheep ranching.

10.7717/peerj.8945/supp-5 Supplemental Information 5 Count data: number of observations, group size and segment coordinates.

10.7717/peerj.8945/supp-6 Supplemental Information 6 Segment data. Location and variables that define each segment.

## Additional Information and Declarations

### Field Study Permissions

The following information was supplied relating to field study approvals (i.e., approving body and any reference numbers): Permission for the research was given by the Direction of Conservation and Protected Areas, and the Direction of Wildlife of the Chubut Province (DF & FS-SSG, Permit 69/2016).

### Data Availability

The following information was supplied regarding data availability: The raw data is available in the Supplemental Files.
