# Supplementary material for: Choosing what is left: the spatial structure of a wild herbivore population within a livestock-dominated landscape
Source: PeerJ. 2020 Apr 8;8:e8945. doi: 10.7717/peerj.8945 (PMC7150538; doi:10.7717/peerj.8945)
Supplement: Supplemental Information 1 [file peerj-08-8945-s001.docx]

**Supplemental Information 1. Analysis of the variation coefficient of the Normalized Vegetation Index (CV NDVI).**

The variation coefficient of the Normalized Vegetation Index (CV NDVI) was calculated in each pixel - of 250 m resolution - from MODIS MOD13Q1 satellite images of the period 2010 to 2014, available at https://lpdaac.usgs.gov. The average values of the CV NDVI in each segment (1.8 x 2 km^2^ see in the main paper the section “*Density surface model (DSM)”*) and in each cell (4 km^2^) of the prediction grid (see in the main paper the section “*Abundance estimation*”) were calculated. The map of the spatial variation of the CV NDVI was constructed (Fig. 1a) and the boundaries of the vegetation units of Península Valdés - defined by Bertiller et al. (2017; Fig. 1b) - were superimposed (Fig. 1a). Then, the mean NDVI CV was calculated in each vegetation unit (Table 1). The behavior of the variable in each stratum was visualized by the 'box-plot' chart (Fig.2), while the significant differences were evaluated by means of Wilcoxon rank sum test (Table 1).
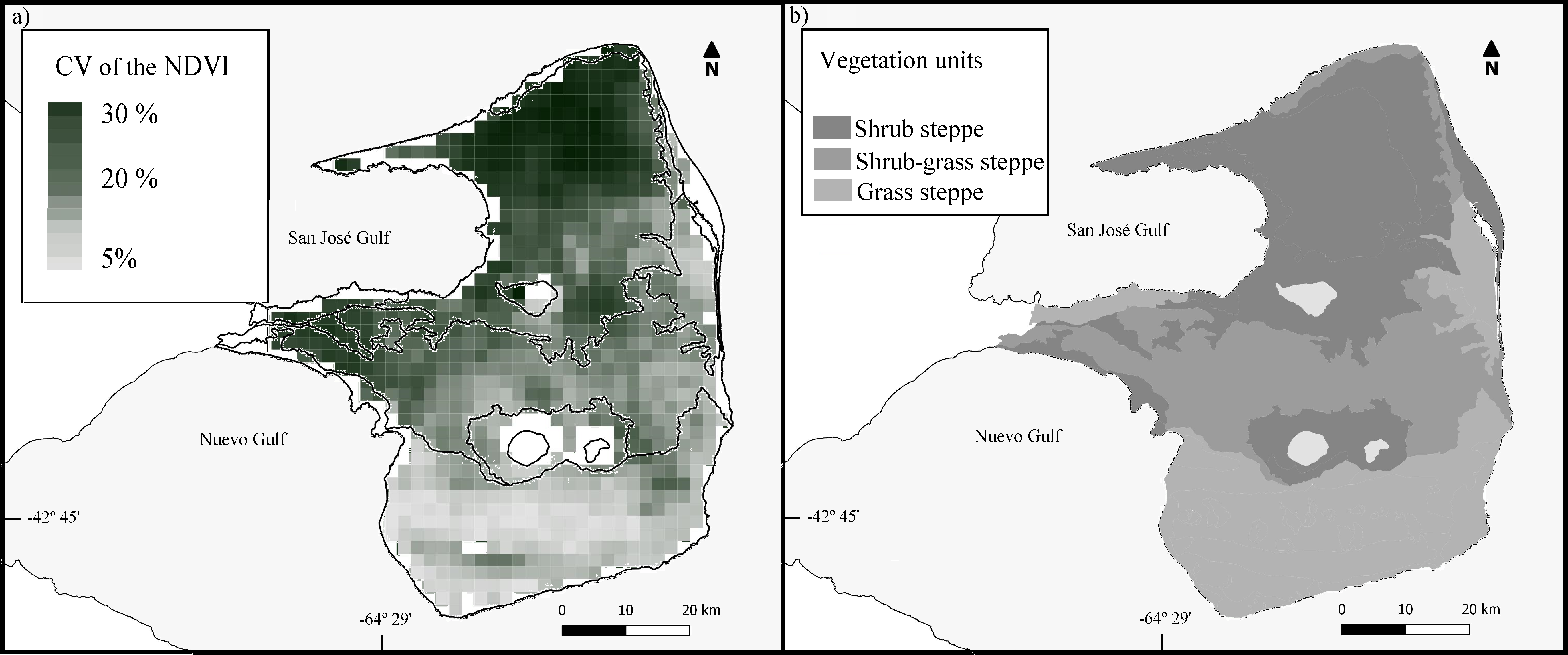


**Figure 1** a) The variation coefficient of the NDVI. The limits of vegetation units are indicated in black. b) Vegetation units of the Peninsula Valdés (modified from Bertiller et al., 2017).

**Table 1.** The variation coefficient of the NDVI by vegetation unit. Different letters indicate significant differences (*P* < 0.005) according to the Wilcoxon rank sum test.

| Vegetation unit | Mean of the CV of the NDVI between 2010 - 2014 | Confidence interval (95%) |
| --- | --- | --- |
| Shrub steppe | 17.9^a^ | 17.61 - 18.26 |
| Grass-shrub steppe | 15.45^b^ | 15.23 - 15.67 |
| Grass steppe | 11.81^c^ | 11.46 - 12.17 |


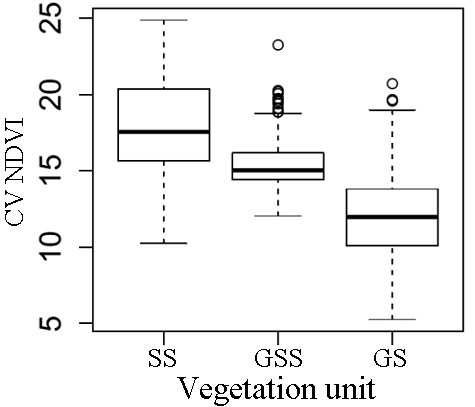


**Figure 2.** Box-plot of the variation coefficient of the Normalized Vegetation Index (CV NDVI) by each vegetation unit: Shrub steppe (SS), grass-shrub steppe (GSS) and grass steppe (GS).
