# Supplementary material for: Choosing what is left: the spatial structure of a wild herbivore population within a livestock-dominated landscape
Source: PeerJ. 2020 Apr 8;8:e8945. doi: 10.7717/peerj.8945 (PMC7150538; doi:10.7717/peerj.8945)
Supplement: Supplemental Information 2 [file peerj-08-8945-s002.docx]

**Supplemental Table 1. Comparison of average densities estimated by each model (PV, SHEEP and NOSHEEP) for the whole PV and in areas with and without sheep ranching.**

According to DSM methodology (Miller et al., 2013), we analyzed the spatial variation in the abundance of guanacos for all the PV area and the contrasting SHEEP – NOSHEEP scenarios. Following the modeling procedure detailed in the main text, we selected the best model according to Miller et al. (2013) criteria (see subsection “*Density surface model (DSM)*” in the main text), identifying the significant variables that account for the spatial variation in the abundance of guanacos in each scenario. Additionally, we estimated guanaco densities for each area by each model (Table 1).

**Table 1.** Average density (guanacos.km^-2^) estimated by PV, SHEEP and NOSHEEP models for the whole Península Valdés (3196 km^2^), and the areas with and without sheep ranching (2616 and 580 km^2^ respectively). The best model selected for each area is indicated in bold.

|  | PV model | SHEEP model | NOSHEEP model |
| --- | --- | --- | --- |
| PV Area | **11.71**  **^(95%CI:9.93-13.82)^** | 14.27  ^(95%CI:10.15-20.05)^ | 32.5  ^(95%IC:20.81-50.75)^ |
| SHEEP Area | 8.58  ^(95%IC:7.2-11.22)^ | **8.02**  **^(95%CI:6.42-10.02)^** | 34.66  ^(95%IC:21.74-55.25^ |
| NOSHEEP Area | 25.86  ^(95%IC:19.49-36.29)^ | 42.43  ^(95%CI: 26.23-68.65)^ | **22.76**  **^(95%IC:15.98-32.41)^** |
