# Supplementary material for: Choosing what is left: the spatial structure of a wild herbivore population within a livestock-dominated landscape
Source: PeerJ. 2020 Apr 8;8:e8945. doi: 10.7717/peerj.8945 (PMC7150538; doi:10.7717/peerj.8945)
Supplement: Supplemental Information 3 [file peerj-08-8945-s003.docx]

**Supplemental Figure 1. Detection function modeling**

As mentioned in the subsection “*Estimating the detection function*”, we fitted a detection function *g(y)* to account for the probability of detecting guanacos by the standard distance sampling methodology (Buckland et al., 1993). For each area evaluated (Península Valdés: PV, and areas with and without sheep ranching: SHEEP and NOSHEEP) we compared three different key functions as candidates, the half-normal, uniform and Hazard rate (Thomas et al., 2010). Possible effects of data truncation (the removal of 10% of the extreme sightings according to Buckland et al., 2001; Thomas et al., 2010; Buckland et al. 2015) were assessed using quantile–quantile plots (Q–Q plots) and the Cramer-von Mises test for the candidate functions. Then, we analyzed the effect of the group size as a covariate, and chose the best model for each area (Fig. 1) following the Akaike information criterion (AIC, Burnham and Anderson 2002), the Q–Q plots and the Cramer-von Mises test. All analyses were performed using the ‘Distance’ package version 0.9.6 (Miller 2017) for R.


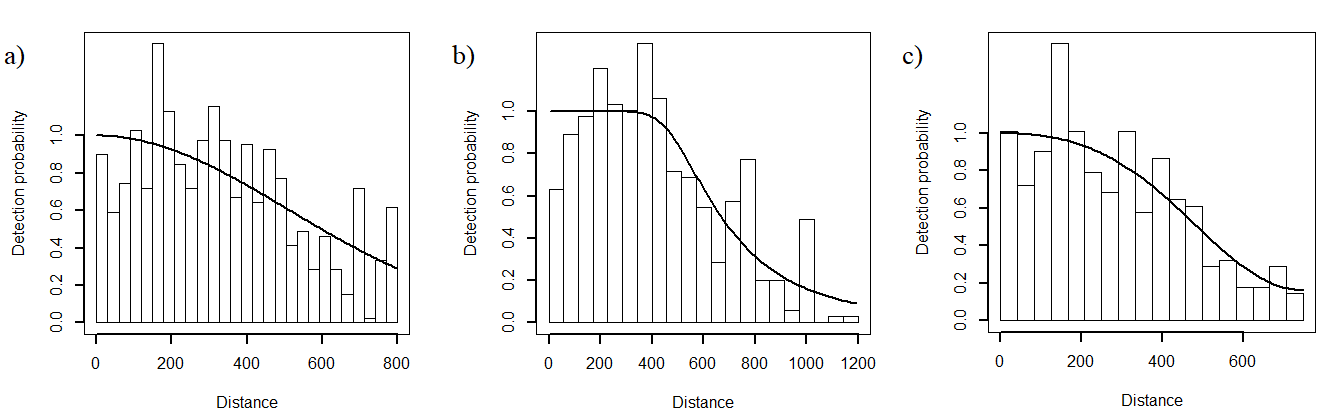


**Figure 1.** **Distribution of perpendicular detection distances of *Lama guanicoe* sightings according to each evaluated area.** Solid line represents the final fitted key functions: a) Half-normal with the truncated data for PV, b) Hazard-rate using group size as a covariate for SH areas, c) Uniform function with the truncated data for NOSH area. The bars represent the observed data grouped into distance intervals according to the perpendicular distance at which they were detected.
