# Supplementary material for: Choosing what is left: the spatial structure of a wild herbivore population within a livestock-dominated landscape
Source: PeerJ. 2020 Apr 8;8:e8945. doi: 10.7717/peerj.8945 (PMC7150538; doi:10.7717/peerj.8945)
Supplement: Supplemental Information 4 [file peerj-08-8945-s004.docx]

**Supplemental Figure 2. Effect of significant smooth terms on the response variable (expressed as number of guanacos per segment), according to the selected models for Península Valdés and the areas with and without sheep ranching.**

The behavior of each significant smooth term is shown in the response scale after the smoother was transformed, and assuming other variables are at their average value.


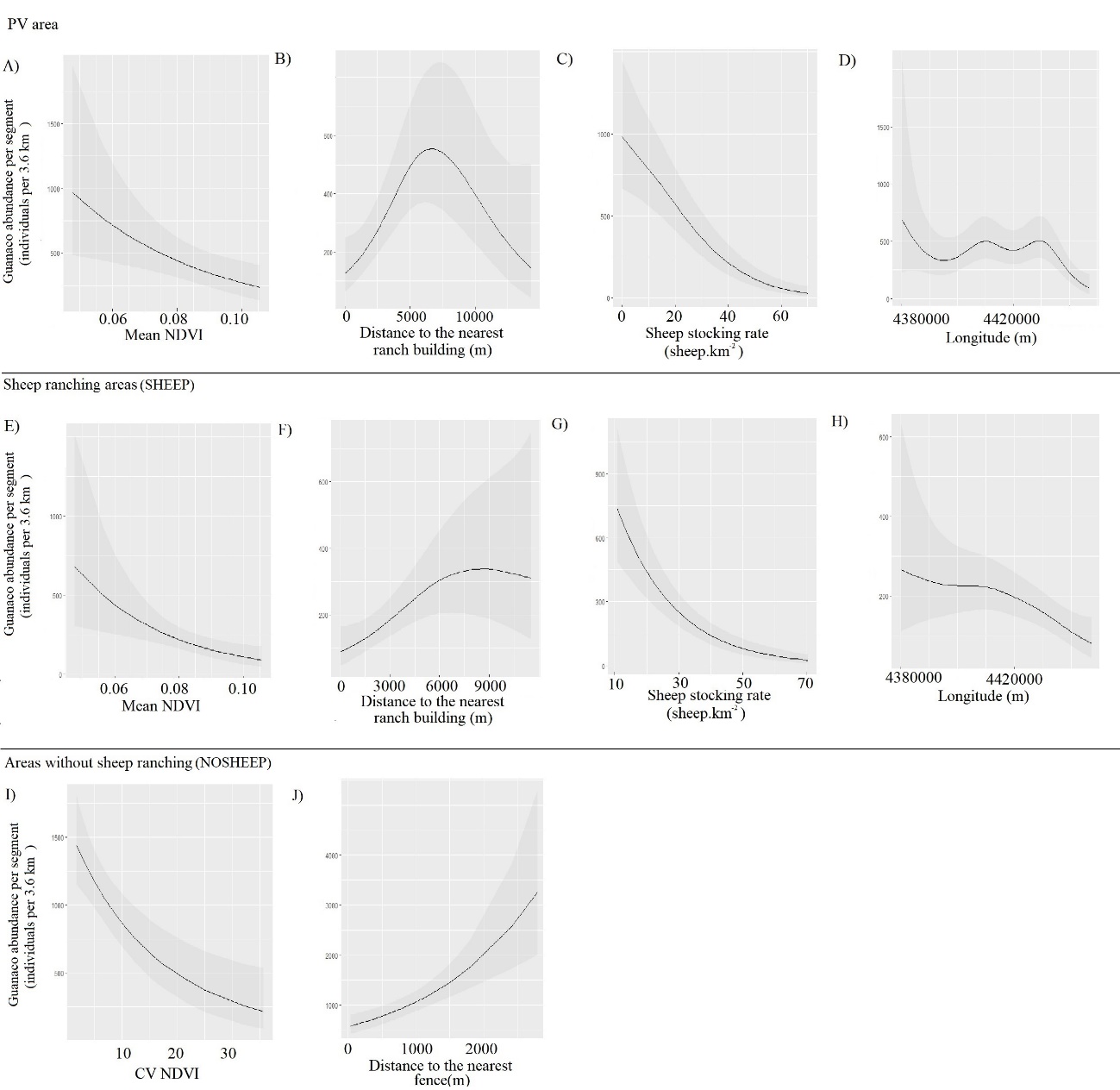


**Figure 2.** Effects of the significant predictors on the abundance of *Lama guanicoe* according to the best-fit model for each area analyzed. Smooth terms are shown in the response scale (i.e. smooth terms were link-transformed). Significant terms for the model of PV area (A, B, C and D), sheep ranching areas (SHEEP; E, F, G and H) and areas where the activity has been ceased (NOSHEEP; I and J). The solid lines represent the estimated smoothing terms (s) of each predictor and the gray shading represents 95% confidence intervals for the mean effect.
